# Rebuilding the atrophied brain: 6-month nasal esketamine therapy expands key frontal and hippocampal regions and reduces serum neurofilament levels in patients with major depressive disorder. A proof-of-concept study of the depTesk (DEPression treatment with ESKetamine) study

**DOI:** 10.1017/S1092852925100631

**Published:** 2025-10-21

**Authors:** Ana Rodríguez Lorente, María Pilar Campos-Navarro, Ángela Gil Montoya, Celia Marín Pérez, María Luisa Maso Navarro, Tomás Orgaz Morales, Nuria López Ramirez, Juan Antonio García-Carmona

**Affiliations:** 1 Department of Psychiatry, Santa Lucía University Hospital, Cartagena, Murcia, Spain; 2 Mental Health Research and Training Unit, Murcia Health Service, Murcia, Spain; 3 Department of Laboratory Medicine, Santa Lucía University Hospital, Cartagena, Murcia, Spain.; 4 Department of Neuroradiology, Santa Lucía University Hospital, Cartagena, Murcia, Spain.; 5 Group of Clinical and Experimental Pharmacology, Institute for Biomedical Research of Murcia (IMIB), Murcia, Spain.; 6 Department of Neurology, Santa Lucía University Hospital, Cartagena, Spain; 7 Faculty of Pharmacy and Nutrition, San Antonio Catholic University of Murcia (UCAM), Murcia, Spain

**Keywords:** Esketamine intranasal, major depression, neurofilaments, brain volume, tractography, fractional anisotropy, mean diffusivity

## Abstract

**Objectives:**

This proof-of-concept study aimed to assess the impact of intranasal esketamine (ESK-IN) in brain volume and neurofilament light chain (sNfL) over 6-months in patients with treatment resistant depression (TDR).

**Methods:**

Seven TRD patients received ESK-IN while continuing oral antidepressants. Clinical evaluations were conducted at baseline, 1, 3, and 6 months, with MRI scans and blood samples taken at baseline and 6 months. Brain volume was assessed using VolBrain2 and DSI studio.

**Results:**

Compared to controls, TRD patients initially showed lower volumes (mm^3^) in key cortical regions such as the insula (p = 0.0156), the frontal lobe (p = 0.0228) the superior parietal lobe (p = 0.0402), both superior (p = 0.0216) and inferior (p = 0.0437) temporal lobes and subcortical regions such as the nucleus accumbens (p = 0.0056), putamen (p = 0.0083), thalamus (p = 0.0102) and the hippocampus (p = 0.0001). Brain volume increased in the frontal cortex (p = 0.0295), the anterior cingulate (p = 0.0496), and hippocampus (p = 0.0015), as well as in the volume and fiber tracts associated with emotional regulation, such as the frontoparahippocampal (p = 0.0156 and p = 0.0313, respectively), the frontoparietal (p = 0.0496 and p = 0.0156, respectively) and the frontal aslant tract after 6 months on treatment with ESK-IN. In parallel, sNfL levels decreased post-treatment, indicating potential neuroprotective effects.

**Conclusions:**

ESK-IN may promote structural changes in regions associated with mood regulation and neuroplasticity, while also reducing neuronal damage in TRD patients.

## Background

Depression is a multifaceted mental health disorder characterized by persistent sadness, loss of interest, and a range of cognitive and physical symptoms, significantly affecting global health systems, economies, and societal functioning. It is a leading cause of disability worldwide, contributing to substantial healthcare costs, loss of productivity, and increased mortality, particularly through suicide.^1^ The neurobiological underpinnings of depression have been extensively studied, with particular attention given to the role of brain-derived neurotrophic factor (BDNF) and structural brain changes. BDNF is a critical neurotrophic factor involved in neurogenesis, synaptic plasticity, and neuronal survival, and its dysregulation has been implicated in the pathophysiology of depression.^2^ ^,^^3^ Recent advances in the treatment of depression have introduced intranasal esketamine (ESK-IN), a rapid-acting antidepressant that has been shown to be effective in treatment-resistant depression (TRD) in both clinical trials and real-world studies.^4^ ^,^^5^ ^,^^6^ Esketamine, an enantiomer of ketamine, exerts its effects primarily through antagonism of the N-methyl-D-aspartate (NMDA) receptor, leading to increased glutamate release and subsequent activation of AMPA receptors.^7^ This mechanism is believed to enhance synaptic plasticity and promote BDNF signaling, which may contribute to its rapid antidepressant effects.^8^ Findings from preclinical research suggest that esketamine may induce structural brain changes, particularly in regions associated with mood regulation, such as the frontal cortex and limbic system.^9^ Nonetheless, specific effects of esketamine on brain volume and BDNF expression in patients with depression remain unexplored. In addition, neurofilament light chain (sNfL) is a protein of the neuronal cytoskeleton and is recognized as a biomarker for disease prognosis and monitoring recurrences, including multiple sclerosis, cognitive decline, stroke, traumatic brain injury, and Guillain–Barré syndrome.^10^ ^,^^11^ ^,^^12^ sNfL levels are highly responsive to microstructural changes at the subclinical level. It remains controversial whether structural brain changes play a role in psychiatric disorders, such as major depressive disorder.^13^ Nonetheless, research about neurofilaments in depression is scarce.^14^ ^,^^15^

Given the novelty of ESK-IN treatment and the limited neuroimaging data available, the present work was designed as a proof-of-concept study from the DEPression Treatment with ESKetamine (depTesk) study, which aimed to investigate the clinical and molecular effects of ESK-IN on brain volume in patients diagnosed with major depression over a 6-month treatment period. Secondary aims were to assess brain atrophy in patients with depression compared to healthy controls and measure blood levels of sNfL as a potential biomarker of depression remission and relapse.

## Methods

### Study design and participants

**Figure 1.**
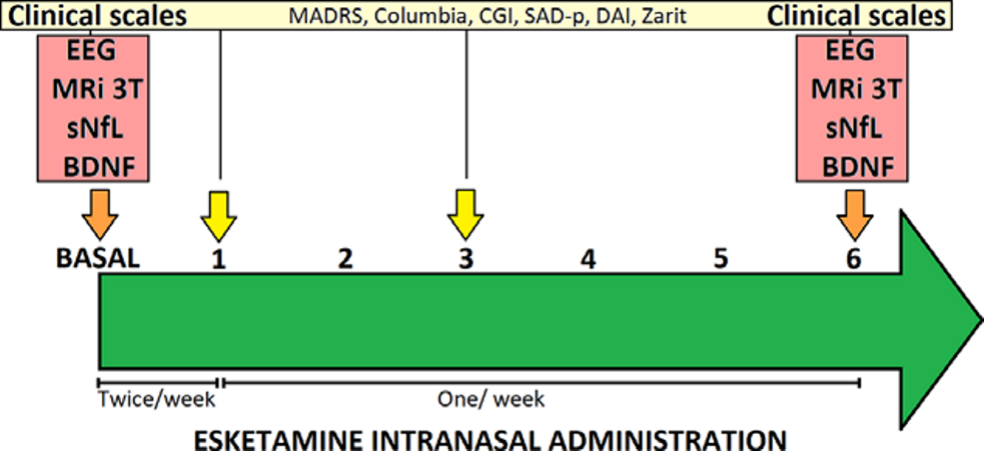
Diagram of the prospective study design, showing treatment, tests, data collection at intervals, and key measurement points.

This pilot study was designed (Figure 1) as a 6-month prospective, open-label trial involving patients diagnosed with major depressive disorder (MDD) who met the criteria for TRD.^16^ Clinical assessments, electroencephalograms, brain neuroimaging, and blood sampling were carried out at baseline (within 7 days prior to esketamine initiation) and after 6 months of ESK-IN treatment. Furthermore, clinical assessments were also performed at 1 and 3 months.

Participants were recruited from outpatient mental health centers and asked to provide informed consent prior to enrollment. The inclusion criteria were as follows: age >18 years, having a confirmed diagnosis of MDD, and having failed to respond to at least two different classes of antidepressants. Exclusion criteria included the following: under 18 years, traumatic brain injury, stroke, multiple sclerosis, a relapse-associated worsening within the last <3 months, uncontrolled epilepsy, congenital brain malformations, and neurodevelopmental disorders and neurodegenerative diseases such as Alzheimer’s disease, amyotrophic lateral sclerosis, Friedreich’s ataxia, Huntington’s disease, dementia with Lewy bodies, Parkinson’s disease and spinal muscular atrophy.

### Treatment protocol and clinical follow-up

Participants received ESK-IN^17^ from their previous oral antidepressant regimen. The treatment protocol involved initial dosing of ESK-IN twice weekly for the first 4 weeks, followed by weekly administration for the next 20 weeks, with a total treatment duration of 6 months. Clinical follow-up was assessed (at baseline, 1, 3, and 6 months using the following scales: 1) the Montgomery–Åsberg Depression Rating Scale (MADRS), 2) the Columbia-Suicide Severity Rating Scale (C-SSRS), 3) the Patient Health Questionnaire-9 (PHQ-9), 4) the SAD persons (SAD-p) Scale, 5) the Disability Assessment Instrument (DAI), and 6) the Zarit Burden Interview (Zarit) Scale. Details about clinical scales can be found in the supplementary material.

### Neuroimaging procedures

Structural magnetic resonance imaging (MRI) was performed at baseline and after six months of adjunctive ESK-IN treatment to assess changes in brain volume. We used a 3 T MR system (Siemens Prisma) to acquire MRI scans, including a 3D T1-weighted (T1w) and diffusion-weighted (DWI) images. A Magnetization Prepared Rapid Gradient Echo (MPRAGE) sequence was used for T1w images (TE = 5.57 ms; TR = 2040 ms; TI = 1100 ms; slice thickness = 0.9 mm; voxel size = 1 mm × 1 mm; 192 slices; flip angle = 8). A multishell diffusion scheme was used for DWI images, which was acquired in an A > > P phase encoding direction, with a repetition time of 3200 msec and an echo time of 90 msec. In addition to *b* = 0 images (five averages), *b*-values were 1000 and 2000 s/mm^2^. The number of diffusion sampling directions was 64 and 64, respectively. The in-plane resolution and slice thickness was 0.9 mm, with a total of 192 slices (no gaps) acquired per participant. To address image distortion, an additional *b* = 0 image with the same dimensions was acquired with opposite phase encoding polarity (P > > A). 3D T1w images were processed and analyzed using the VolBrain2 software, while DWI images were processed and analyzed using the DSI studio software. VolBrain2 is an automated, web-based tool designed for the segmentation and volumetric analysis of brain structures from MRI scans.^18^ The volume of 140 brain regions and subregions was analyzed (details in the supplementary material). DSI Studio is a specialized software suite for processing DWI data and performing tractography to map brain white matter pathways.^19^ ^,^^20^ White matter was analyzed with metrical data such as volume (mm^3^), length, and number of tracts, and its microstructure, indexed as fractional anisotropy (FA) and mean diffusivity (MD), was then measured from DWI images and extracted from DSI studio analysis. FA is a measure of the degree of anisotropy, or directional dependence, of a diffusion process in a biological tissue, commonly used to measure the microstructural properties of tissues, such as white matter tracts in the brain. FA decreases with demyelination, inflammation, edema, or axonal loss.^21^ MD reflects the overall degree of diffusional freedom in the tissue and can be used to characterize the structural properties of the tissue. MD is considered an indirect marker of microstructural integrity.^22^ The main 20 association and projection fascicles and commissural tracts were analyzed (details in the supplementary material).

### Assessment of brain atrophy in depression

Patient 3D T1w MRI scans were acquired at our institution, while control data were obtained from the OpenNeuro repository, an international public neuroimaging database. For each patient, three healthy controls were selected, matched for age, sex, and race, due to their known influence on brain volume. All control scans were acquired on 3 T MRI scanners (GE Discovery MR750 and Philips Achieva dStream) with protocols comparable (including slice thickness = 0.9 mm; voxel size = 1 × 1 mm; 192 slices) to those used for patients, including those in used previous studies.^23^ ^,^^24^ Control and patient images were subsequently processed using the VolBrain2 software as described above. These measures were implemented to minimize potential confounding in case–control comparisons.

### Neurofilament analysis

Blood samples were collected from participants at baseline and after 6 months of treatment to measure sNfL levels using the Lumipulse G1200 automated system with the Lumipulse G NfL blood kit (Ref: 81215, Fujirebio Inc., Tokyo) according to the manufacturer’s instructions. The functional LLOQ for sNfL concentration was 3.25 pg/mL. Two types of quality control samples provided in the kit were measured before the serum samples. All measured values were within the calibration range.^25^

### Statistical analysis

All analyses were performed using the software IBM SPSS Statistics version 21.0 (IBM Corp., Armonk, NY). Quantitative variables were expressed as means [standard error of the mean], and categorical variables as frequencies (percentages). Data were analyzed using appropriate statistical methods to compare case–control, baseline, and post-treatment measures. The Friedman test was used to analyze clinical tests. Case–control analyses (brain volume) were performed using the Mann–Whitney U test, while pre- and post-treatment analyses (brain volume, tractography, sNfL levels) were performed using the Wilcoxon signed-rank test. Given the large number of comparisons (>70 nuclei and fascicles), multiple comparisons were controlled using the Benjamini–Hochberg false discovery rate (FDR) procedure, with a significance threshold set at q < 0.05. Results are reported both as raw p-values and as FDR-adjusted q-values. Complete tables with the values for each nucleus are provided in the supplementary material. Differences with a p-value <0.05 were considered significant and shown with the FDR adjustment as q-value.

### Ethical considerations

The authors assert that all procedures contributing to this work comply with the ethical standards of the relevant national and institutional committees on human experimentation and with the Helsinki Declaration of 1975, as revised in 2008 and approved by the Santa Lucia University Hospital Ethics Committee (CEI.24-02_ESK). Before study initiation, written informed consent covering retrospective, screening, and prospective data collection was obtained from participants. The protocol and consent forms were approved by the respective institutional ethics committee.

## Results

### Demographics and clinical characteristics

**Figure 2.**
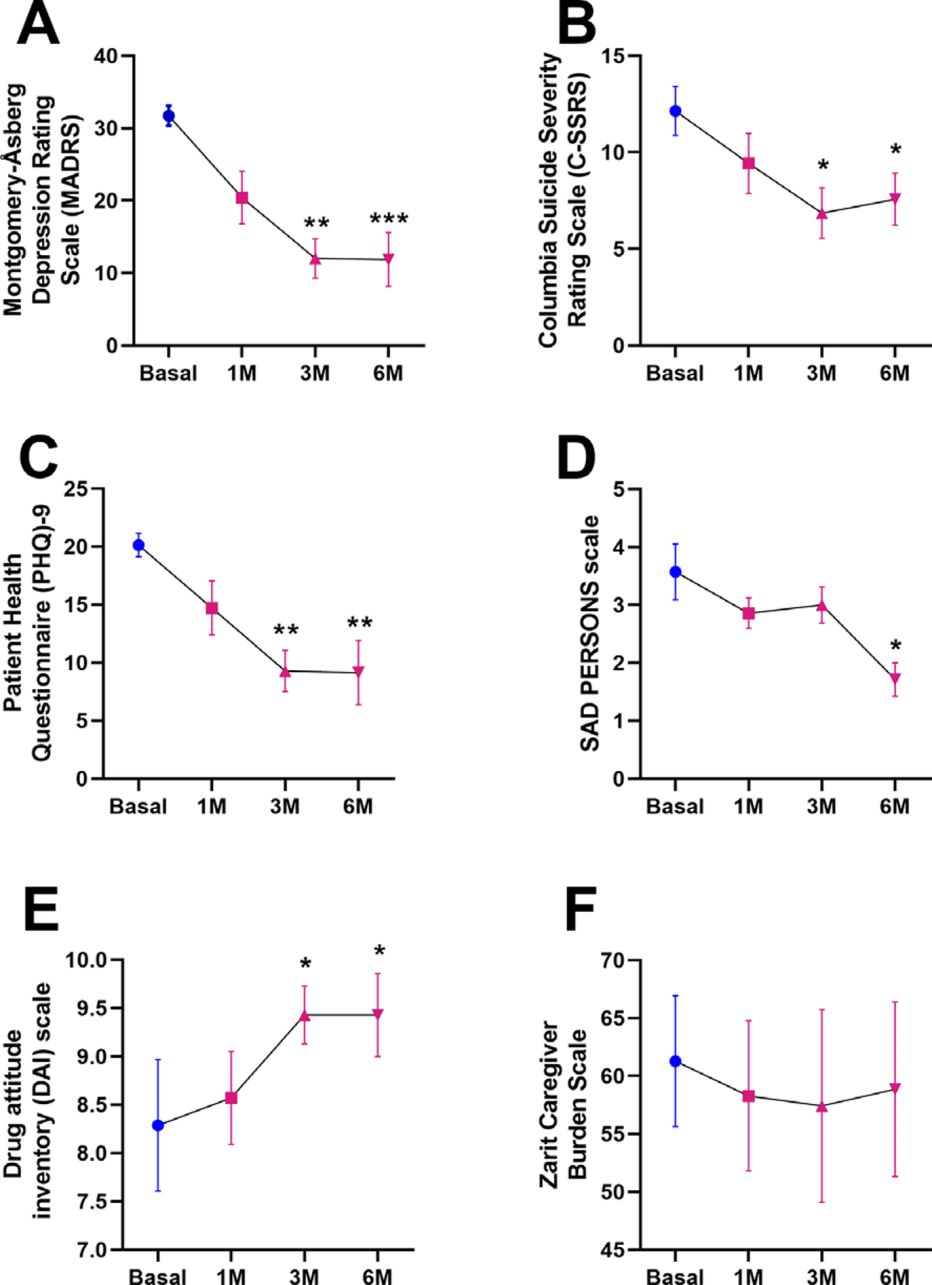
Clinical scales before and up to 6 months after esketamine treatment. (A) MADRS, (B) C-SSRS, (C) PHQ-9, (D) SAD-p Scale, (E) DAI, and (F) Zarit Scale. Data are presented as mean ± SEM. Non-parametric repeated measures Friedman test comparing score changes at the first, third, and sixth months with baseline values before initiating esketamine. ***p < 0.001, **p < 0.01, and *p < 0.05 versus baseline.

A total of 7 participants completed the 6-month treatment protocol and were included in this preliminary analysis from the depTesk study; their basic sociodemographic characteristics are presented in Table 1. The sample consisted of 5 women (71%) and 2 men (29%), with a mean age of 48.2 ± 4.8 years. Most of the patients exhibited previous suicidal behavior (71%), had a previous major depressive episode (MDE, 86%), and had other comorbid psychiatric disorders, such as personality (29%), eating (29%) or substance use (SUD, 29%) disorders. At baseline, participants exhibited moderate to severe depressive symptoms and suicidal ideation (Figure 2A–D), as assessed by the MADRS (31.71 ± 1.35), PHQ-9 (20.14 ± 1.01), SAD-p Scale (3.57 ± 0.48), and C-SSRS (12.14 ± 1.26). Patients’ attitudes toward medication (Figure 2E) achieved a score of 8.28 ± 0.67 in the DAI, and caregivers’ burden (Figure 2F) achieved a score of 61.29 ± 5.64 in the Zarit Scale, both at baseline. The Friedman test indicated that treatment with esketamine showed significant improvements at 3 and 6 months in the MADRS (Figure 2A, 12.00 ± 2.71, p = 0.0081 and 11.86 ± 3.71, p = 0.0006, respectively), C-SSRS (Figure 2B, 7.57 ± 1.34, p = 0.0389, only at 6 months), PHQ-9 (Figure 2C, 9.28 ± 1.76, p = 0.004 and 9.14 ± 2.75, p = 0.0028, respectively), SAD-p Scale (Figure 2D, 1.71 ± 0.28, p = 0.0113, only at 6 months), and DAI (Figure 2E, 9.43 ± 0.29, p = 0.0334 and 9.43 ± 0.42, p = 0.0389, respectively) scores. No significant improvements were found in caregiver burden by the Zarit Scale (Figure 2F) at 3 (57.43 ± 8.31, p = 0.3069) and 6 months (58.86 ± 7.54, p = 0.3935).

**Table 1.** Demographic and Clinical Data

	Cohort N = 7
**Sex (%)**	
Women	5 (71)
Men	2 (29)
**Age (y ± SEM)**	48.2 ± 4.8
**Education (%)**	
Primary	4 (57)
Secondary	2 (29)
University	1 (14)
**Marital status (%)**	
Single	3 (42)
Married/coupled	2 (29)
Divorced	2 (29)
**Living (%)**	
Alone	2 (29)
With parents	2 (29)
With own family	2 (29)
With friends	1 (14)
**Employed (%)**	1 (14)
**Comorbid psychiatric diagnoses (%)**	
None	3 (42)
Eating disorder	1 (14)
Personality disorder	2 (29)
SUD	1 (14)
**Previous suicidal behavior**	5 (71)
**Number of previous MDEs**	
0	1 (14)
1	2 (29)
2	2 (29)
3	2 (29)
**Age at first MDE (y ± SEM)**	34.2 ± 4.2
**Number of previous antidepressant treatments**	
3	1 (14)
4	4 (57)
5	2 (29)
**Duration of current MDE (y ± SEM)**	2.8 ± 0.5
**Concomitant treatments at baseline**	
Number of antidepressants	
2	4 (57)
3	3 (42)
Mood stabilizer	3 (42)
Number of antipsychotics	
1	2 (29)
2	3 (42)
Number of benzodiazepines	
1	2 (29)
2	4 (57)
3	-
4	1 (14)
Beta-blocker	1 (14)

Abbreviations: AP, antipsychotic; MDE, major depression episode; SUD, substance use disorder; y, years.

### Effects of 6-month esketamine treatment on neurofilament serum levels

**Figure 3.**
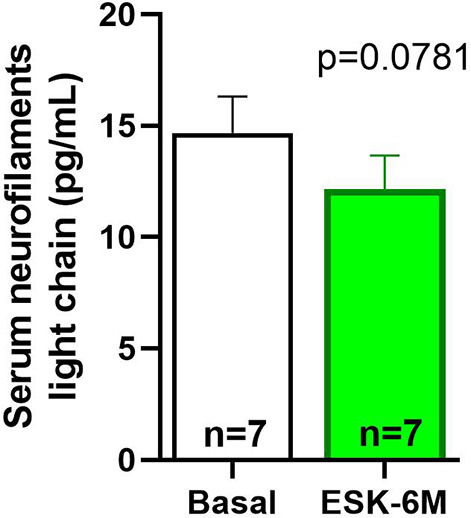
Neurofilament light chain (NfL) levels. Data are presented as mean ± SEM. Wilcoxon signed-rank test comparing NfL after 6 months of treatment with esketamine.

As shown in Figure 3, Wilcoxon signed-rank test showed a statistical trend toward lower sNfL levels after 6 months of treatment with esketamine (14.67 ± 1.64 versus 12.14 ± 1.51, p = 0.0781).

### Brain atrophy in depression

**Figure 4.**
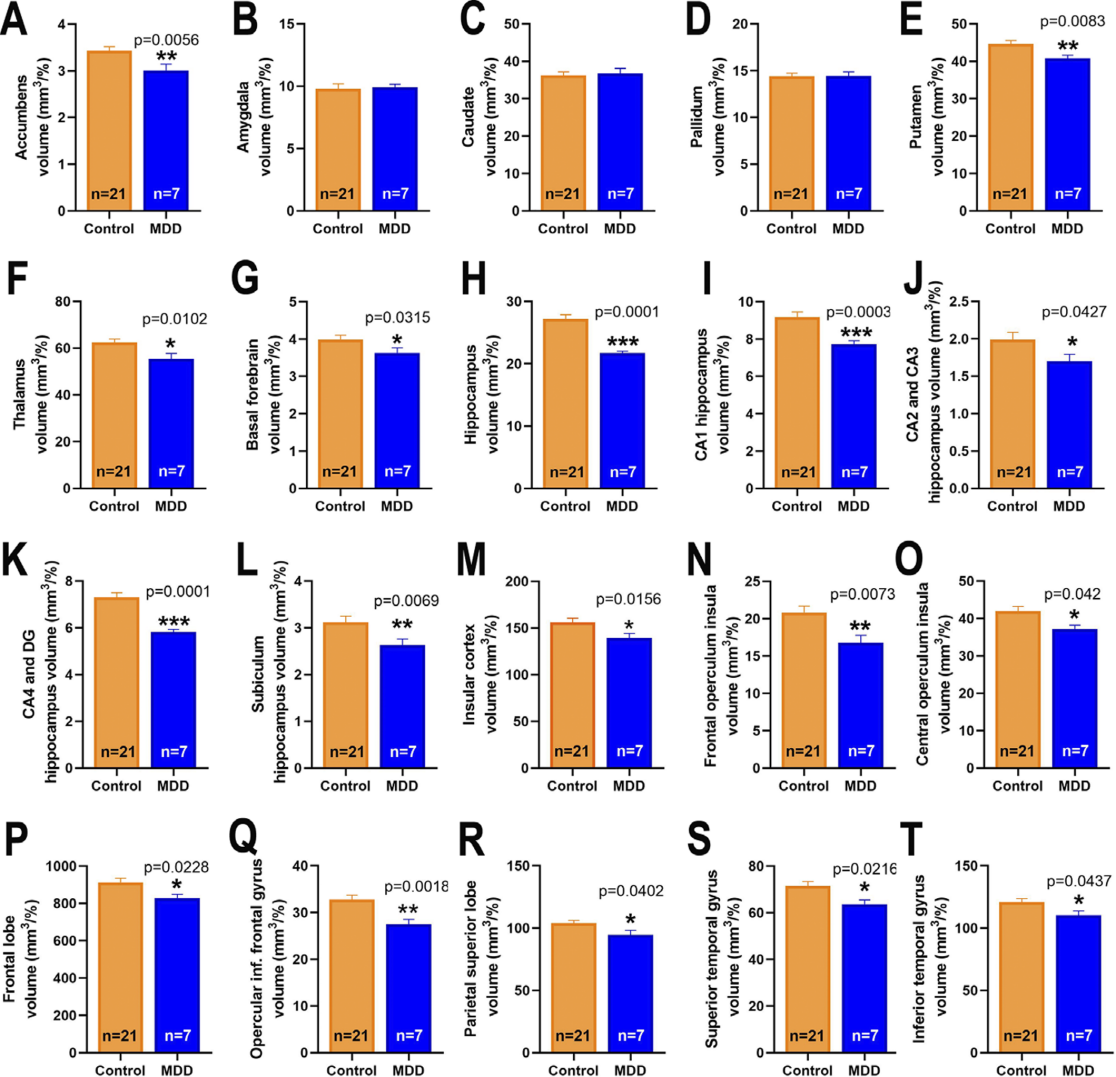
Brain region volume comparison between patients with depression and healthy controls. (A) accumbens, (B) amygdala, (C) caudate, (D) pallidum, (E) putamen, (F) thalamus, (G) basal forebrain, (H) hippocampus, (I) CA1, (J) CA2-CA3, (K) CA4-DG, (L) subiculum, (M) insular cortex, (N) frontal operculum insula, (O) central operculum insula, (P) frontal lobe, (Q) opercular inferior frontal gyrus, (R) parietal superior lobe, (S) superior temporal gyrus, and (T) inferior temporal gyrus. Data are presented as mean ± SEM. Mann–Whitney U test comparing brain region volumes between the depression group (MDD) before initiating esketamine with matched healthy controls. ***p < 0.001, **p < 0.01, and *p < 0.05.

The statistical analysis with the Mann–Whitney U test of the data obtained from the VolBrain2 analysis of the MRI scans on subcortical brain structures comparing healthy 21 matched controls and our 7 TRD patients revealed a significantly decreased volume (mm^3^) of nucleus accumbens (3.00 ± 0.14 *versus* 3.43 ± 0.08, U = 94.50, p = 0.0056, FDR-adjusted q = 0.0373; Figure 4A), putamen (40.75 ± 0.82 *versus* 44.63 ± 0.93, U = 98.50, p = 0.0083, FDR-adjusted q = 0.0534; Figure 4E), thalamus (55.36 ± 2.33 *versus* 62.42 ± 1.41, U = 101, p = 0.0102, FDR-adjusted q = 0.0601; Figure 4F), basal forebrain (3.63 ± 0.14 *versus* 3.99 ± 0.12, U = 116, p = 0.0315, FDR-adjusted q = 0.0042; Figure 4G), and hippocampus (21.69 ± 0.32 *versus* 27.20 ± 0.66, U = 14.50, p = 0.0001, FDR-adjusted q = 0.0025; Figure 4H). Similar differences were found in all the hippocampus subregions (Figure 4I–L): CA1 (7.65 ± 0.17 *versus* 9.25 ± 0.28, U = 29.50, p = 0.0003, FDR-adjusted q = 0.0017), CA2-CA3 (1.70 ± 0.09 *versus* 2.00 ± 0.09, U = 63.50, p = 0.0427, FDR-adjusted q = 0.0545), CA4-DG (5.82 ± 0.10 *versus* 7.31 ± 0.19, U = 10.50, p = 0.0001, FDR-adjusted q = 0.0012), and subiculum (2.63 ± 0.13 *versus* 3.11 ± 0.13, U = 48.50, p = 0.0069, FDR-adjusted q = 0.0266). No differences were found in amygdala, caudate, and pallidum subcortical regions (Figure 4B–D). The analysis also found a significantly reduced volume in cortical regions, such as the insula (139.4 ± 4.63 *versus* 156.3 ± 4.36, U = 106, p = 0.0156, FDR-adjusted q = 0.0425; Figure 4M) and its operculofrontal (16.78 ± 0.99 *versus* 20.84 ± 0.87, U = 97, p = 0.0073, FDR-adjusted q = 0.0282) and operculocentral (37.13 ± 1.04 *versus* 41.89 ± 1.34, U = 119.5, p = 0.042, FDR-adjusted q = 0.0697) regions (Figure 4N,O), frontal lobe (824.6 ± 19.20 *versus* 912.1 ± 23.57, U = 111, p = 0.0228, FDR-adjusted q = 0.0276; Figure 4P), frontal opercular region (27.15 ± 1.09 *versus* 32.73 ± 0.99, U = 82, p = 0.0018, FDR-adjusted q = 0.0039; Figure 4Q), superior parietal lobe (94.35 ± 3.81 *versus* 104.1 ± 2.06, U = 119, p = 0.0402, FDR-adjusted q = 0.1432; Figure 4R), and both superior (63.64 ± 1.77 *versus* 71.43 ± 1.91, U = 110.5, p = 0.0216, FDR-adjusted q = 0.1206) and inferior (109.3 ± 3.32 *versus* 120.8 ± 2.83, U = 120, p = 0.0437, FDR-adjusted q = 0.1426) temporal lobes (Figure 4S,T) in MDD patients compared to the control group.

### Effects of 6-month esketamine treatment in brain volumetry and tractography

**Figure 5.**
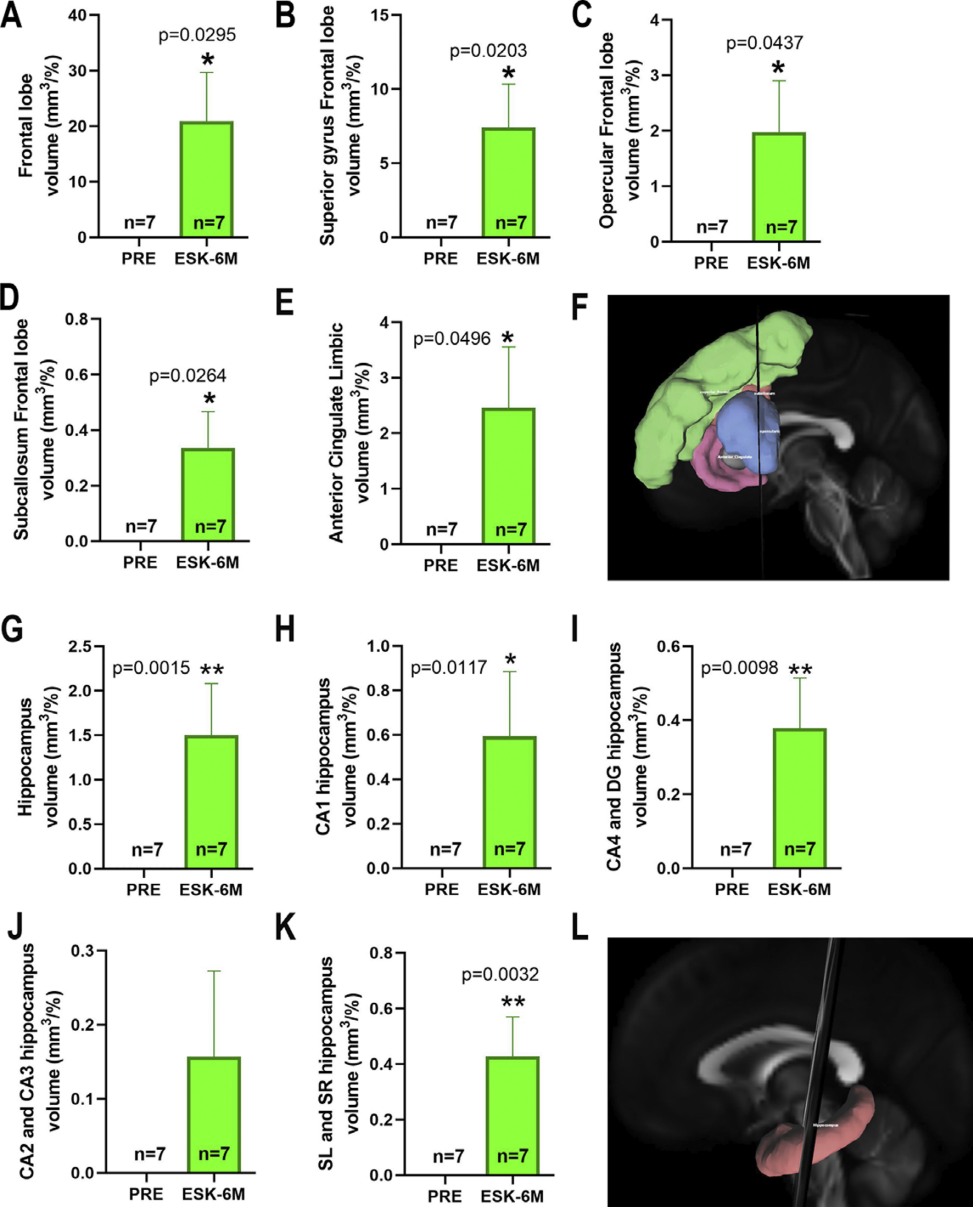
Effect of esketamine on brain volume. (A) frontal lobe, (B) superior frontal gyrus, (C) frontal operculum, (D) subcallosal frontal lobe, (E) anterior cingulate of the limbic system, (F) tridimensional model including previous regions, (G) hippocampus, (H) CA1 region, (I) CA4-DG regions, (J) CA2-CA3 regions, (K) subiculum, and (L) hippocampus tridimensional model. Data are presented as mean ± SEM. Wilcoxon signed-rank test comparing the effect of esketamine after 6 months (ESK-6) with baseline (PRE) values before starting esketamine. **p < 0.01, *p < 0.05 versus baseline.

**Figure 6.**
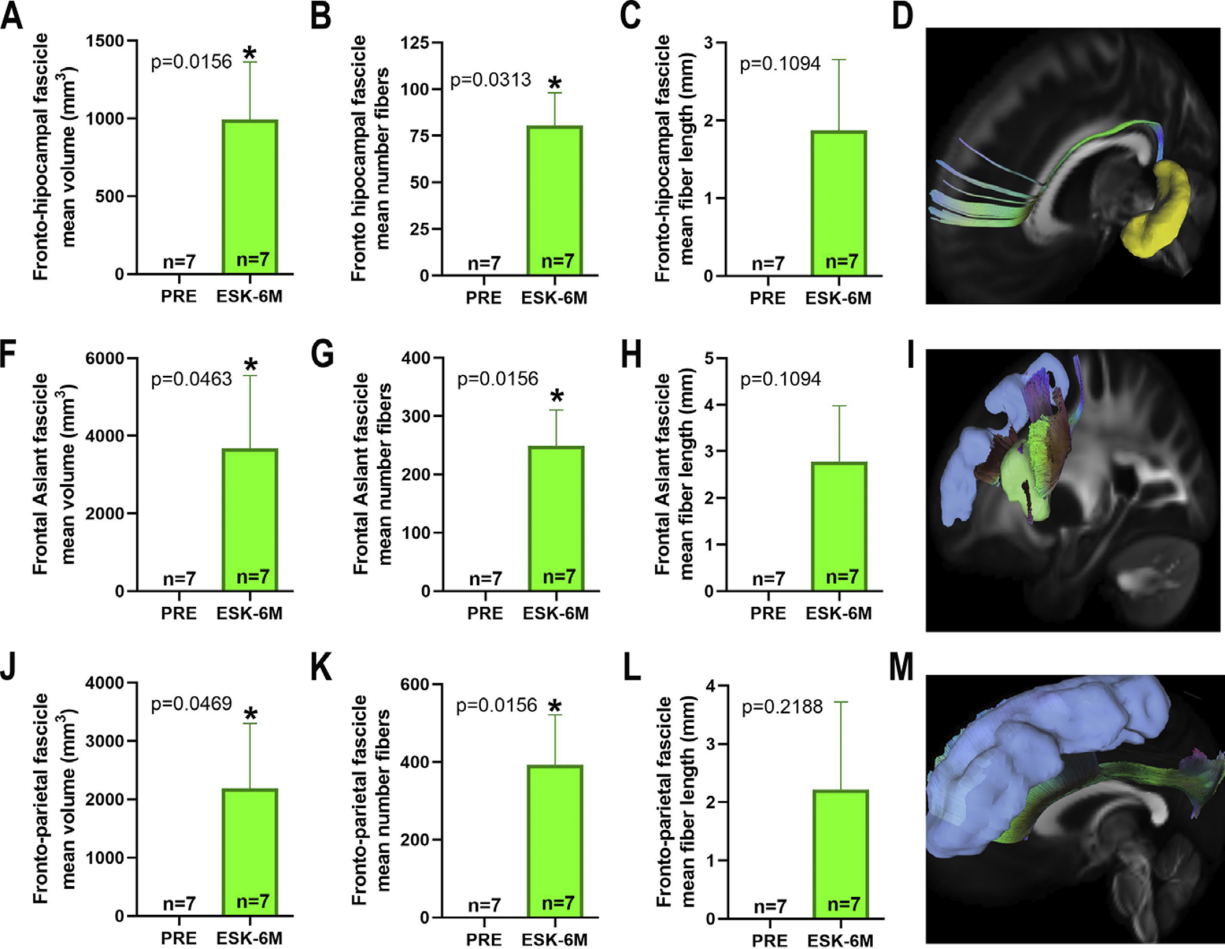
Effect of esketamine on brain tractography measured as volume, length, and fascicle tracts. (A–D) fronto-parahippocampal tract, (F–I) frontal aslant tract, and (J–M) frontoparietal tract. Data are presented as mean ± SEM. Wilcoxon signed-rank test comparing esketamine at 6 months (ESK-6 M) with baseline values before starting esketamine (PRE). *p < 0.05 versus baseline.

**Figure 7.**
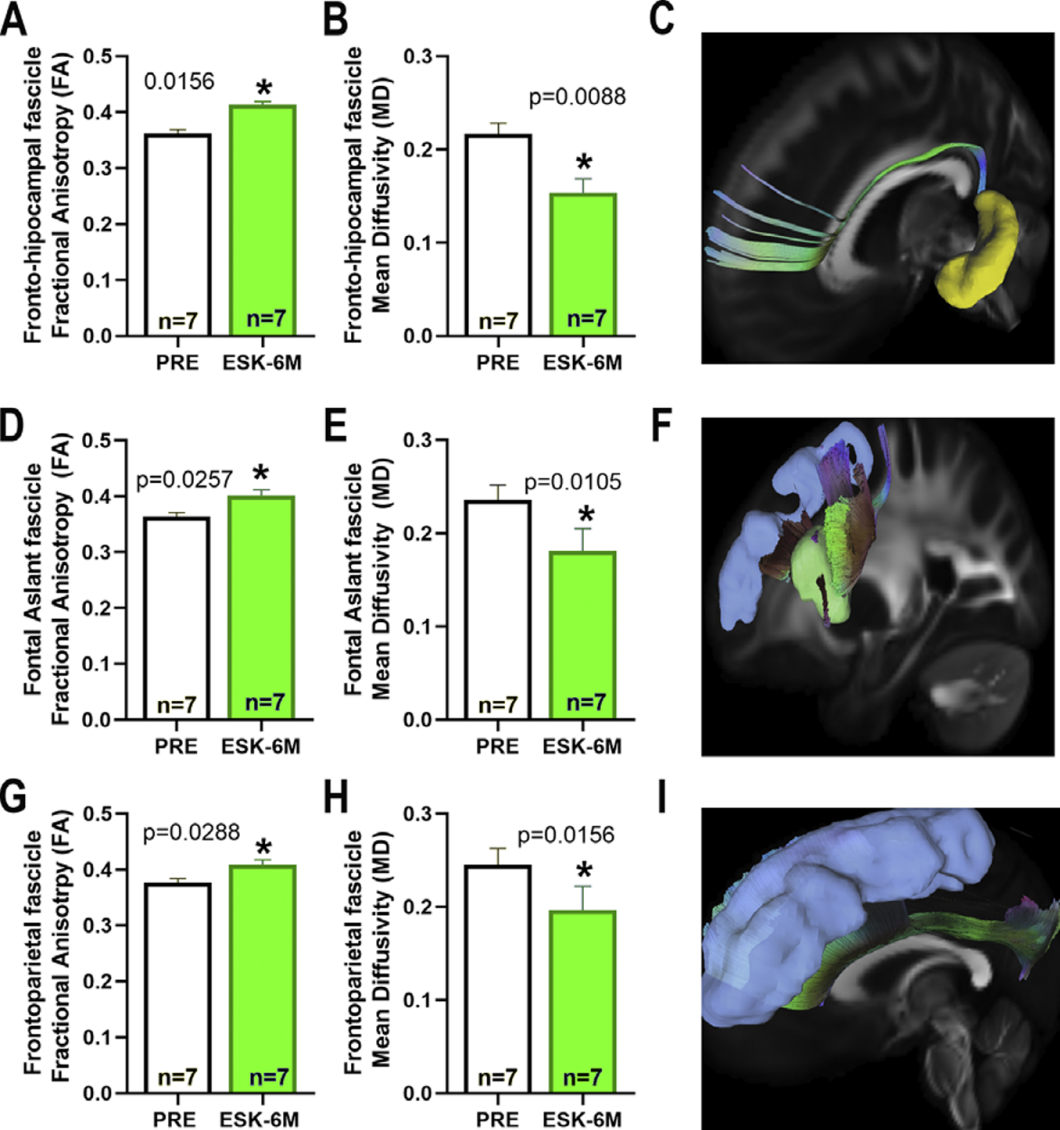
Effect of esketamine on brain diffusion tensor imaging measured as fractional anisotropy (FA) and mean diffusivity (MD). (A–C) fronto-parahippocampal tract, (D–F) frontal aslant tract, and (G–I) frontoparietal tract. Data are presented as mean ± SEM. Wilcoxon signed-rank test comparing esketamine at 6 months (ESK-6 M) with baseline values before initiating esketamine (PRE). *p < 0.05 versus baseline.

The statistical analysis with the Wilcoxon rank pairs test of the data obtained from the VolBrain2 analysis of the pre-ESK-IN and 6 months post-ESK-IN MRI scans on cortical and subcortical brain structures revealed a significantly increased volume (mm^3^) of the frontal lobe (Figure 5A, 20.86 ± 8.13, p = 0.0159, FDR-adjusted q = 0.0626), superior frontal gyrus (Figure 5B, 7.39 ± 2.93, p = 0.0164, FDR-adjusted q = 0.0428), frontal operculum (Figure 5C, 1.98 ± 0.92, p = 0.0036, FDR-adjusted q = 0.0686), subcallosal frontal gyrus (Figure 5D, 0.34 ± 0.13, p = 0.0017, FDR-adjusted q = 0.1188), limbic anterior cingulate (Figure 5E, 2.46 ± 1.09, p = 0.0033, FDR-adjusted q = 0.2810), and hippocampus (Figure 5G, 1.50 ± 0.57, p = 0.0015, FDR-adjusted q = 0.0375) and its subregions CA1 (Figure 5H, 0.59 ± 0.29, p = 0.0017, FDR-adjusted q = 0.0514), CA4-DG (Figure 5I, 0.38 ± 0.13, p = 0.0016, FDR-adjusted q = 0.0784) and SL/SR (Figure 5K, 0.43 ± 0.14, p = 0.0014, FDR-adjusted q = 0.0416). Furthermore, the tractography analysis of the main association and commissural fascicles showed a significant increase in both fascicle volume and the number of detected fibers in the fronto-parahippocampal (Figure 6A,B) (991.3 ± 371.2, p = 0.0156 and 80.57 ± 17.55, p = 0.0313, respectively), frontoparietal (Figure 6F,G) (2189 ± 1110, p = 0.0496 and 392.1 ± 128.5, p = 0.0156, respectively), and frontal aslant (Figure 6J,K) (4014 ± 1688, p = 0.0469 and 248.7 ± 61.68, p = 0.0156, respectively) fascicles after 6 months of treatment with ESK-IN. Nonetheless, no differences were found in fascicle length (Figure 6C,H,L). Similarly, after 6 months of ESK-IN treatment, significant changes in FA and MD were observed within the fronto-hippocampal (Figure 7A–C) (FA pre: 0.362 ± 0.006 versus post: 0.414 ± 0.005, p = 0.0156; and MD pre: 0.216 ± 0.011 versus post: 0.154 ± 0.015, p = 0.0088, respectively), FAT (Figure 7D–F) (FA pre: 0.364 ± 0.006 versus post: 0.401 ± 0.010, p = 0.0257; and MD pre: 0.235 ± 0.016 versus post: 0.181 ± 0.024, p = 0.0105, respectively), and frontoparietal (Figure 7G–I) fascicles (FA pre: 0.357 ± 0.007 versus post: 0.409 ± 0.008, p = 0.0156; and MD pre: 0.245 ± 0.017 versus post: 0.196 ± 0.026, p = 0.0088, respectively).

## Discussion

In this study, we present the protocol and initial data from the ongoing depTesk study. The preliminary findings provide compelling evidence that 6-month treatment with ESK-IN is effective in patients with MDD, leading to a significant increase in brain volume in frontal, limbic, and hippocampal regions. This effect appears to be associated with decreased sNfL levels, suggesting reduced neuronal damage.

The profile of our 7 patients is similar to those in other real-world studies: women, ~50 years old, first episode of depression at ~35–40 years, comorbid personality (10–15%) or SUD disorders (6–25%), previous 3–4 depressive episodes, current MDE duration 1–2 years, 9–25% showing suicidal behavior, and treatment with 3–4 antidepressant trials lifetime with concomitant mood stabilizers (23%), benzodiazepines (43%) and antipsychotics (46%).^5^ ^,^^6^ ^,^^26^ ^,^^27^ ^,^^28^ ^,^^29^ ^,^^30^ ^,^^31^ According to previous reports, we found significant clinical improvements with ESK-IN at 3 and 6 months of treatment. As previously shown, clinical responders and remission rates increased between the first and third months.^6^ Moreover, patients with TRD who experienced remission or response after 4 months of treatment with ESK-IN showed clinical superiority compared with patients treated with antidepressant plus placebo.^32^ Evidence indicates that the benefit of esketamine extends beyond acute use, with substantial improvements in the chances of achieving remission over other treatment strategies after 6 months.^33^

Furthermore, our results showed a significantly lower brain volume (both cortical, mainly in the frontal lobe and insula, and subcortical, mainly in the hippocampus) in TRD patients receiving oral antidepressants compared to matched healthy controls. These findings are in line with previous data from meta-analyses that reported widespread gray-matter deficits from the anterior cingulate, medial prefrontal and orbitofrontal cortices, insula, hippocampus, parietal, and temporal regions in recurrent MDD.^34^ ^,^^35^ Furthermore, a recent study demonstrated that compared to healthy controls, MDD patients with preserved gray- and white-matter volumes in all regions responded better to treatment with SSRI antidepressants, while MDD patients with subtle widespread decreased volumes were non-responders.^36^ Altogether, these findings suggest that TRD pathophysiology could be different from other types of depression and that it could be associated with brain atrophy as a marker of non-response to SSRI treatment.

Previous studies demonstrated that ketamine increases the volume of the hippocampus in healthy subjects^37^ and in animal models of depression.^38^ Furthermore, findings from animal models of depression demonstrated that esketamine increases dendritic spine density and maturation in the prefrontal cortex.^39^ For the first time in TRD patients, we showed that 6-month adjunctive treatment with ESK-IN increases the volume of some brain regions such as the frontal lobe, in particular superior gyrus, operculum and subcallosal regions, limbic anterior cingulate, and hippocampus. MRI DTI showed increased volume and number of tracts in the fronto-parahippocampal, frontal aslant, and frontoparietal fascicles. Accordingly, FA values increased significantly while MD decreased, indicating an improvement in microstructural integrity following treatment, consistent with enhanced fiber organization. It is worth noting that some of these brain regions, such as the prefrontal cortex, medial and middle frontal regions, and cingulate gyrus, have been reported to be associated with reduced FA in treatment-naïve MDD patients.^40^ ^,^^41^ ^,^^42^ Furthermore, it has been reported that MD decreases in the hippocampus after electroconvulsive therapy in TRD patients.^43^ ^,^^44^

The fronto-parahippocampal or uncinate fasciculus, which connects the prefrontal cortex with the hippocampus, is implicated in memory and emotional regulation, and disruptions in this tract may contribute to cognitive and emotional disturbances commonly observed in depression.^45^ ^,^^46^ Disruptions in this tract may contribute to anhedonia, a key symptom of depression, particularly in patients who report a diminished ability to experience pleasure in activities they once enjoyed.^46^ ^,^^47^ The frontal aslant tract, which connects the frontal cortex and the operculum, plays a role in executive functions and speech fluency. Its impairment may reflect deficits in cognitive flexibility, speech abnormalities, including “robotic speech,” delayed response times, and automatic crying, wherein individuals may find themselves crying without clear emotional triggers.^48^ Finally, the frontoparietal fasciculus, linking prefrontal and parietal regions, is crucial for maintaining attention and spatial processing. Studies evaluating brain connectivity in depression are scarce, be it cross-sectional or case–control.^49^ ^,^^50^ Current research indicates that MDD is associated with altered functional connectivity within and between key brain networks, including the default mode network (DMN), the frontoparietal network (FPN), and the salience network (SN).^51^ These networks connect several critical regions such as the cingulate cortex, prefrontal cortex, anterior cingulate, parietal cortex, and insula, as well as the fronto-parahippocampal, frontal aslant, and frontoparietal fasciculi. It is hypothesized that changes in the neural tracts identified through DTI may underlie these functional connectivity alterations. Nonetheless, the current fragmented nature of clinical and neuroimaging data hinders a comprehensive and systematic evaluation of this hypothesis.^50^ There is only one study evaluating the role of esketamine in brain connectivity, which reported no differences in white matter fibers after 2 weeks of treatment with i.v. esketamine.^52^

Furthermore, we found that patients treated with ESK-IN had lower sNfL levels after 6 months. In this regard, previous studies showed that sNfL levels in MDD patients (but not in schizophrenia) were significantly higher than reference values of the healthy population.^53^ The high inter-individual variability of sNfL levels and the lack of neurobiological understanding of its release are some of the major current limitations.^19^

In conclusion, this pilot study suggests that the addition of ESK-IN to ongoing antidepressant treatment may be associated with increased brain volume in the frontal and limbic regions in patients with depression. These results highlight the potential of esketamine as a transformative treatment option for individuals with TRD and underscore the importance of neuroplasticity and neurotrophic signaling in the pathophysiology of depression. Nonetheless, while these preliminary findings are promising, they should be interpreted with caution given the small sample size and warrant replication in larger, controlled studies.

## Limitations

First, a main limitation of this study is the relatively small sample size, which restricts the statistical power and limits the generalizability of our findings. This issue is particularly relevant when examining volumetric changes across multiple brain regions, as the reliability of such findings may be affected by the limited number of participants. Therefore, the results should be interpreted with caution and considered preliminary in nature. In this regard, the present work should be regarded as a proof-of-concept or pilot study that provides an initial basis for future investigations with larger and more representative samples. Second, it is worth noting that an important limitation of the present study is the lack of a control group receiving either ESK-IN treatment or placebo, which prevents us from definitively establishing whether the observed brain changes are specific to esketamine. However, it should be noted that all patients were already receiving oral antidepressant treatment several months prior to study participation, and baseline assessments therefore reflect the effects of ongoing pharmacotherapy. The subsequent increase in brain volume observed after 6 months occurred only after the addition of esketamine, suggesting that these changes may not be attributable to standard antidepressant treatment alone. Nevertheless, potential placebo effects cannot be completely ruled out and should be taken into account when interpreting the findings. Third, the possible influence of concomitant medications (benzodiazepines, mood stabilizers, antipsychotics) on neuroimaging outcomes is also a limitation. Although all participants were maintained on stable pharmacological regimens throughout the study period or even concomitant medication was decreased or discontinued between baseline and follow-up scans, we cannot fully exclude the possibility that medication effects contributed to the observed findings. Nevertheless, the within-subject design minimizes this confounding factor, as each participant served as their own control. Finally, despite careful matching and use of comparable 3 T MRI protocols, we cannot fully exclude the possibility of residual differences in acquisition parameters or other unmeasured factors that might influence case–control comparisons. Therefore, results involving healthy controls should be interpreted with caution.

## Supporting information

Rodríguez Lorente et al. supplementary material Rodríguez Lorente et al. supplementary material

## Data Availability

The data are available from the authors upon reasonable request.

## Supplementary material

The supplementary material for this article can be found at http://doi.org/10.1017/S1092852925100631.
