# Supplementary material for: Rebuilding the atrophied brain: 6-month nasal esketamine therapy expands key frontal and hippocampal regions and reduces serum neurofilament levels in patients with major depressive disorder. A proof-of-concept study of the depTesk (DEPression treatment with ESKetamine) study
Source: CNS Spectr. 2025 Oct 21;30(1):e83. doi: 10.1017/S1092852925100631 (PMC13064687; doi:10.1017/S1092852925100631)
Supplement: Rodríguez Lorente et al. supplementary material [file S1092852925100631sup001.docx]

**Supplementary material**

**Clinical scales**

The Montgomery-Åsberg Depression Rating Scale (MADRS) is a clinician-administered scale used to assess the severity of depressive symptoms. It consists of 10 items that measure aspects such as mood, feelings of guilt, insomnia, and suicidal thoughts. Scores range from 0 to 60, with higher scores indicating more severe depression. A score of 0–6 is considered minimal depression, 7–19 mild, 20–34 moderate, and 35 or above severe, 2) the Columbia-Suicide Severity Rating Scale (C-SSRS) is a clinician-administered scale that assesses the severity and intensity of suicidal ideation and behavior. It includes questions on the frequency, duration, and intensity of suicidal thoughts and attempts. Scores are used to categorize risk levels, with higher scores indicating greater severity of suicidal thoughts or actions, 3) the Patient Health Questionnaire-9 (PHQ-9) is a validated, self-administered screening tool designed to assess the presence and severity of depressive symptoms based on the diagnostic criteria of the DSM-IV for major depressive disorder. It consists of nine items, each corresponding to a core symptom of depression, which are rated on a 4-point Likert scale ranging from 0 ("not at all") to 3 ("nearly every day"), yielding a total score between 0 and 27. The interpretation of the PHQ-9 score allows clinicians to classify depression severity as minimal (0–4), mild (5–9), moderate (10–14), moderately severe (15–19), or severe (20–27), 4) the SAD persons (SAD-p) scale is a brief, clinician-administered tool used to assess the risk of suicide in individuals. It includes 10 items that evaluate risk factors such as sex, age, depression, previous suicide attempts, and social supports. Each item is scored as either 0 or 1, resulting in a total score between 0 and 10. Higher scores indicate higher suicide risk. The scale helps in identifying individuals who may require more intensive monitoring or intervention. A score of 0-2 suggests low risk, 3-4 moderate risk, and 5 or more indicates high risk, 5) the Disability Assessment Instrument (DAI) is a tool used to measure functional disability across various domains, including self-care, mobility, and social functioning. It provides a score reflecting the patient's level of disability, where higher scores indicate greater impairment in daily living activities. The instrument is often used to evaluate the impact of medical conditions or treatments on a patient’s functional abilities and 6) Zarit Burden Interview (Zarit) is a caregiver-report scale designed to assess the burden experienced by those caring for individuals with chronic illnesses, such as dementia. It consists of 22 items evaluating the physical, emotional, and financial strain on caregivers. Scores range from 0 to 88, with higher scores indicating greater caregiver burden.

**VolBrain2 brain volumetric analysis**

VolBrain2 allows for precise extraction and analysis of gray matter, white matter, and subcortical structures providing quantitative measurements of brain volumes. This software is widely used for studies involving brain development, aging, and neurodegenerative conditions. We analyzed the following brain structures: Accumbens, Amygdala, Basal Forebrain, Caudate, Hippocampus (CA1, CA2-CA3, CA4-DG, SR-SL-SM, Subiculum), Pallidum, Putamen, Thalamus; Frontal lobe: Frontal pole, Gyrus rectus Opercular inf. frontal gyrus Orbital inf. frontal gyrus Triangular inf. frontal gyrus Medial frontal cortex Middle frontal gyrus Anterior orbital gyrus Lateral orbital gyrus Medial orbital gyrus Posterior orbital gyrus Precentral gyrus Precentral gyrus medial segment, Subcallosal area, Sup. frontal gyrus, Sup. frontal gyrus medial segment, Supplementary motor cortex, Temporal lobe: Fusiform gyrus, Planum polare, Planum temporale, Inf. temporal gyrus, Middle temporal gyrus, Sup. temporal gyrus, Transverse temporal gyrus, Temporal pole, Parietal lobe: Angular gyrus, Postcentral gyrus, Postcentral gyrus medial segment, Precuneus, Sup. parietal lobule, Supramarginal gyrus, Occipital lobe: Calcarine cortex, Cuneus, Lingual gyrus, Occipital fusiform gyrus, Inf. occipital gyrus, Middle occipital gyrus, Sup. occipital gyrus, Occipital pole, Limbic cortex: Entorhinal area, Anterior cingulate gyrus, Middle cingulate gyrus, Posterior cingulate gyrus, Parahippocampal gyrus, Insular cortex: Anterior insula, Posterior insula, Central operculum, Frontal operculum, Parietal operculum.

**DSI studio tractography analysis**

It supports advanced analyses such as whole-brain connectomics, fiber tracking, and the generation of probabilistic tractography maps. DSI Studio can also perform advanced statistical analyses and visualization of brain networks, providing insights into the structural connectivity of the brain. We analyzed the main brain association and commissural fascicles including the arcuate, the uncinate, the frontoparietal, the frontoparahippocampal, the frontal aslant tract (FAT), the alveus, both the superior and inferior longitudinal fasciculus, the corpus callosum, the anterior, posterior and hippocampal commissures.

**Case-control analysis with FDR (Benjamini–Hochberg) correction for multiple comparisons**

| **TEST** | **Brain region** | **p-value** | **Rank** | **q (=testXp/rank)** |
| --- | --- | --- | --- | --- |
| 24 | Hippocampus CA4-DG | 0.0001 | 2 | 0.0012 |
| 25 | Hippocampus | 0.0001 | 1 | 0.0025 |
| 26 | Hippocampus SLSR | 0.0001 | 3 | 0.0009 |
| 22 | Hippocampus CA1 | 0.0003 | 4 | 0.0017 |
| 11 | Frontal operculum | 0.0018 | 5 | 0.0040 |
| 40 | Accumbens | 0.0056 | 6 | 0.0373 |
| 27 | Hippocampus subiculum | 0.0069 | 7 | 0.0266 |
| 31 | Insula frontopercularis | 0.0073 | 8 | 0.0283 |
| 58 | Putamen | 0.0083 | 9 | 0.0535 |
| 59 | Thalamus | 0.0102 | 10 | 0.0602 |
| 30 | Insula | 0.0156 | 11 | 0.0425 |
| 67 | Temporal superior | 0.0216 | 12 | 0.1206 |
| 33 | Insula posterior | 0.0224 | 13 | 0.0569 |
| 17 | Frontal Lobe | 0.0228 | 14 | 0.0277 |
| 2 | Basal Forebrain | 0.0315 | 15 | 0.0042 |
| 57 | Parietal superior lobe | 0.0402 | 16 | 0.1432 |
| 29 | Insula central operculum | 0.0409 | 17 | 0.0698 |
| 23 | Hippocampus CA2-3 | 0.0427 | 18 | 0.0546 |
| 62 | Temporal inferior | 0.0437 | 19 | 0.1426 |
| 15 | Frontal precentral | 0.0641 | 20 | 0.0481 |
| 56 | Parietal supramarginal gyrus | 0.0703 | 21 | 0.1875 |
| 49 | Occipital superior gyrus | 0.0724 | 22 | 0.1613 |
| 34 | Limbic anterior cingulate | 0.0815 | 23 | 0.1205 |
| 10 | Frontal motor supplementary | 0.0816 | 24 | 0.0340 |
| 4 | Frontal anterorbital gyrus | 0.0865 | 25 | 0.0138 |
| 5 | Frontal cortex medial | 0.1084 | 26 | 0.0208 |
| 32 | Insula occipital operculum | 0.1115 | 27 | 0.1321 |
| 68 | Temporal transverse | 0.1177 | 28 | 0.2858 |
| 36 | Limbic | 0.1245 | 29 | 0.1546 |
| 53 | Parietal cuneus | 0.1246 | 30 | 0.2201 |
| 43 | Occipital fusiform | 0.1311 | 31 | 0.1818 |
| 18 | Frontal subcallosum | 0.1379 | 32 | 0.0776 |
| 41 | Occipital calcarine | 0.1455 | 33 | 0.1808 |
| 61 | Temporal fusiform | 0.1532 | 34 | 0.2749 |
| 63 | Temporal middle | 0.1571 | 35 | 0.2828 |
| 65 | Temporal planum temporale | 0.2150 | 36 | 0.3882 |
| 16 | Frontal precentral segmentum | 0.2356 | 37 | 0.1019 |
| 28 | Insula anterior | 0.2410 | 38 | 0.1776 |
| 66 | Temporal pole | 0.2467 | 39 | 0.4175 |
| 60 | Temporal Lobe | 0.2578 | 40 | 0.3867 |
| 8 | Frontal medial orbital gyrus | 0.2809 | 41 | 0.0548 |
| 35 | Limbic entorhinal | 0.3114 | 42 | 0.2595 |
| 52 | Parietal Lobe | 0.3242 | 43 | 0.3921 |
| 39 | Limbic posterior cingulate | 0.3377 | 44 | 0.2993 |
| 21 | Frontal pars triangularis | 0.3512 | 45 | 0.1639 |
| 19 | Frontal superior gyrus | 0.3585 | 46 | 0.1481 |
| 20 | Frontal medial superior gyrus | 0.3652 | 47 | 0.1554 |
| 48 | Occipital polare | 0.3940 | 48 | 0.3940 |
| 64 | Temporal planum polare | 0.4636 | 49 | 0.6055 |
| 37 | Limbic middle cingulate | 0.4637 | 50 | 0.3431 |
| 1 | Amygdala | 0.4795 | 51 | 0.0094 |
| 44 | Occipital inferior gyrus | 0.4883 | 52 | 0,4132 |
| 12 | Frontal orbital | 0.5134 | 53 | 0.1162 |
| 55 | Parietal medial postcentral gyrus | 0.6490 | 54 | 0.6610 |
| 7 | Frontal laterorbital gyrus | 0.6683 | 55 | 0.0851 |
| 13 | Frontal polare | 0.6779 | 56 | 0.1574 |
| 14 | Frontal posterior orbital gyrus | 0.7071 | 57 | 0.1737 |
| 51 | Parietal angular | 0.,722 | 58 | 0.6349 |
| 38 | Limbic parahipoccampal | 0.7268 | 59 | 0.4681 |
| 54 | Parietal postcentral | 0.7464 | 60 | 0.6718 |
| 46 | Occipital lingus | 0.7571 | 61 | 0.5709 |
| 42 | Occipital cuneus | 0.7870 | 62 | 0.5331 |
| 6 | Frontal gyrus rectus | 0.9004 | 63 | 0.0858 |
| 45 | Occipital Lobe | 0.9007 | 64 | 0.6333 |
| 50 | Pallidum | 0.9212 | 65 | 0.7086 |
| 3 | Caudate | 0.9422 | 66 | 0.0428 |
| 47 | Occipital medial gyrus | 0.9947 | 67 | 0.6978 |
| 9 | Frontal middle gyrus | 0.9999 | 68 | 0.1323 |

**Pre- post esketamine analysis with FDR (Benjamini–Hochberg) correction for multiple comparisons**

| **TEST** | **Brain region** | **p-value** | **Rank** | **q (=testXp/rank)** |
| --- | --- | --- | --- | --- |
| 63 | Hippocampus | 0.0015 | 1 | 0.0945 |
| 67 | Hippocampus SLSR | 0.0014 | 2 | 0.0469 |
| 66 | Hippocampus CA4-DG | 0.0016 | 3 | 0.0352 |
| 22 | Frontal subcallosum | 0.0017 | 4 | 0.0093 |
| 64 | Hippocampus CA1 | 0.0017 | 5 | 0.0217 |
| 53 | Limbic anterior cingulate | 0.0033 | 6 | 0.0291 |
| 11 | Frontal operculum | 0.0036 | 7 | 0.0056 |
| 8 | Frontal Lobe | 0.0159 | 8 | 0.0159 |
| 23 | Frontal superior gyrus | 0.0164 | 9 | 0.0419 |
| 58 | Insula anterior | 0.0417 | 10 | 0.2418 |
| 57 | Insula | 0.0813 | 11 | 0.4212 |
| 9 | Frontal polare | 0.0820 | 12 | 0.0615 |
| 15 | Frontal middle gyrus | 0.0917 | 13 | 0.1058 |
| 45 | Occipital lingus | 0.0938 | 14 | 0.3015 |
| 26 | Temporal Lobe | 0.0979 | 15 | 0.1696 |
| 21 | Frontal precentral segmentum | 0.1489 | 16 | 0.1954 |
| 20 | Frontal precentral | 0.1531 | 17 | 0.1801 |
| 49 | Occipital superior gyrus | 0.1563 | 18 | 0.4254 |
| 61 | Insula frontopercularis | 0.1741 | 19 | 0.5589 |
| 16 | Frontal anterorbital gyrus | 0.1766 | 20 | 0.1412 |
| 32 | Temporal superior | 0.1777 | 21 | 0.2707 |
| 38 | Parietal medial postcentral gyrus | 0.2188 | 22 | 0.3779 |
| 68 | Hippocampus subiculum | 0.2288 | 23 | 0.6764 |
| 51 | Limbic | 0.2329 | 24 | 0.4949 |
| 33 | Temporal transverse | 0.2351 | 25 | 0.3103 |
| 17 | Frontal laterorbital gyrus | 0.2671 | 26 | 0.1746 |
| 35 | Parietal Lobe | 0.2676 | 27 | 0.3468 |
| 48 | Occipital medial gyrus | 0.2683 | 28 | 0.4599 |
| 55 | Limbic posterior cingulate | 0.2736 | 29 | 0.5188 |
| 31 | Temporal middle | 0.2958 | 30 | 0.3056 |
| 43 | Occipital calcarine | 0.3013 | 31 | 0.4179 |
| 13 | Frontal pars triangularis | 0.3184 | 32 | 0.1293 |
| 41 | Parietal supramarginal gyrus | 0.3438 | 33 | 0.4271 |
| 65 | Hippocampus CA2-3 | 0.3591 | 34 | 0.6865 |
| 34 | Temporal pole | 0.3692 | 35 | 0.3586 |
| 59 | Insula posterior | 0.3853 | 36 | 0.6314 |
| 27 | Temporal fusiform | 0.4375 | 37 | 0.3192 |
| 46 | Occipital fusiform | 0.4487 | 38 | 0.5431 |
| 14 | Frontal cortex medial | 0.4531 | 39 | 0.1626 |
| 44 | Occipital cuneus | 0.4688 | 40 | 0.5156 |
| 50 | Occipital polare | 0.4688 | 41 | 0.5717 |
| 19 | Frontal posterior orbital gyrus | 0.4810 | 42 | 0.2175 |
| 25 | Frontal motor supplementary | 0.4861 | 43 | 0.2826 |
| 7 | Thalamus | 0.4897 | 44 | 0.0779 |
| 5 | Pallidum | 0.5281 | 45 | 0.0588 |
| 56 | Limbic parahipoccampal | 0.5726 | 46 | 0.6970 |
| 62 | Insula occipital operculum | 0.5903 | 47 | 0.7786 |
| 10 | Frontal gyrus rectus | 0.6226 | 48 | 0.1297 |
| 28 | Temporal planum polare | 0.6250 | 49 | 0.3571 |
| 24 | Frontal medial superior gyrus | 0.6586 | 50 | 0.3161 |
| 2 | Amygdala | 0.7241 | 51 | 0.0283 |
| 37 | Parietal postcentral | 0.7508 | 52 | 0.5342 |
| 30 | Temporal inferior | 0.7703 | 53 | 0.4360 |
| 4 | Caudate | 0.7896 | 54 | 0.0584 |
| 60 | Insula central operculum | 0.7955 | 55 | 0.8678 |
| 39 | Parietal cuneus | 0.8125 | 56 | 0.5658 |
| 54 | Limbic middle cingulate | 0.8271 | 57 | 0.7835 |
| 36 | Parietal angular | 0.8438 | 58 | 0.5237 |
| 42 | Occipital Lobe | 0.8673 | 59 | 0.6174 |
| 6 | Putamen | 0.9329 | 60 | 0.0932 |
| 29 | Temporal planum temporale | 0.9375 | 61 | 0.4456 |
| 52 | Limbic entorhinal | 0.9492 | 62 | 0.7961 |
| 3 | Basal Forebrain | 0.9575 | 63 | 0.1455 |
| 12 | Frontal orbital | 0.9697 | 64 | 0.1818 |
| 1 | Accumbens | 0.9999 | 65 | 0.1503 |
| 18 | Frontal medial orbital gyrus | 0.9999 | 66 | 0.2727 |
| 40 | Parietal superior lobe | 0.9999 | 67 | 0.5969 |
| 47 | Occipital inferior gyrus | 0.9999 | 68 | 0.6911 |
